# Mental health of nurses involved with COVID-19 patients in Japan, intention to resign, and influencing factors

**DOI:** 10.1097/MD.0000000000026828

**Published:** 2021-08-06

**Authors:** Takashi Ohue, Eiichi Togo, Yuka Ohue, Kazuko Mitoku

**Affiliations:** Department of Nursing, Faculty of Nursing, Hyogo University, Kakogawa-shi, Hyogo, Japan.

**Keywords:** COVID19, intention to resign, Japanese nurse, mental health

## Abstract

The purpose of this study was to investigate the association between mental health (posttraumatic stress disorder, depression, anxiety disorder, and burnout) and intention to resign, and influencing factors regarding nurses involved with COVID-19 patients in A Prefecture as subjects.

The design is a cross-sectional questionnaire-based study.

Methods are conducted between August 4 and October 26, 2020. Basic attributes (gender, age, years of experience, etc.) were examined. Patient Health Questionnaire-9 and the Generalized Anxiety Disorder-7, Impact of Event Scale-Revised, Maslach Burnout Inventory, “intent to resign,” were used to collect data from nurses working at hospitals treating patients with COVID-19 in Japan.

As a result, between 20% and 30% of nurses involved with patients with COVID-19 are in a state of high mental distress. Regarding the associations between psychiatric symptoms and intention to resign, “I want to quit being a nurse” was affected by “cynicism” and “professional efficacy”; “I want to change hospitals/wards” was affected by “cynicism”; and “subthreshold depression,” “anxiety disorder,” and “burnout” affected “I want to continue working as a nurse.” The increase in the number of patients with COVID-19 was a factor affecting mental health and intention to resign. When the number of patients increased, anxiety disorders and intention to resign also increased. Damage from harmful rumors increased the severity of every psychiatric symptom. To prepare for a pandemic such as COVID-19, it is necessary in normal times to construct psychological support systems and community systems to prevent damage from harmful rumors.

## 1 Introduction

In December 2019, a respiratory disease due to the novel coronavirus was first reported in Wuhan, China. Subsequently, the World Health Organization (WHO) confirmed it as a “public health emergency of international concern,” and on March 11, 2020, declared it a pandemic.

Since the end of December 2019, the media has reported on a new pneumonia caused by COVID-19, which has been spreading domestically and internationally. On January 30, 2020, WHO convened an emergency meeting, and declared the global outbreak of COVID-19 a public health emergency of international concern.^[1]^ In Japan, as of May 19, 2020, 16,365 people have been infected and 763 people have died. On May 6, the International Council of Nurses (ICN) stated that at least 90,000 healthcare workers worldwide had been infected with COVID-19.^[2]^ Reportedly, because of the continuing shortages of items such as infection prevention masks in healthcare settings, the actual number of infections among healthcare workers is possibly twice that. In Japan as well, infections among healthcare workers have been reported. Healthcare workers care for infected patients while simultaneously feeling the fear of becoming infected and dying. In Japan, geographic disparities exist; for example, there are many infections in the large urban centers of the Tokyo metropolitan area, Osaka Prefecture, and Hyogo Prefecture, where nurses are busy caring for patients with COVID-19. Prior studies on infectious diseases reported that during the SARS outbreak in 2003, healthcare workers were afraid of infecting their families, friends, and colleagues, and of being infected themselves. Consequently, they demonstrated high levels of work stress, anxiety, and subthreshold depression.^[3]^ As such, in the current critical situation, mental health disorders among nurses involved with COVID-19 patients are a concern.

There have been reports worldwide about the mental health of nurses involved with COVID-19 patients. In China, which was on the forefront of treating the disease, Jianbo Lai, Simeng Ma, Ying Wang, et al reported on the mental health status of healthcare workers due to COVID-19.^[4]^ Their results showed that healthcare workers in Wuhan experienced a high psychological load, and in particular, female nurses and frontline healthcare workers exhibited mental health problems such as posttraumatic stress disorder (PTSD), depression, anxiety disorders, and sleep disorders. Surveys have been conducted in various countries on the mental health status of nurses involved with COVID-19 patients. For example, in Portugal, when COVID-19 emerged, nurses exhibited higher levels of depression, anxiety, and stress than the general Portuguese population group. Overall, it was reported that nurses who felt that personal protective equipment was inadequate in quantity and quality exhibited significantly high levels of depression, anxiety, and stress.^[5]^

In Japan, the results of a survey conducted by the Japanese Nursing Association indicated that in 15.4% of the responding hospitals, nurses and practical nurses had resigned, giving as reasons changes in the working environment and risk of infection in conjunction with the spread of COVID-19 infection. Once these responses were narrowed down to publicly designated medical institutions for infectious diseases and cooperating medical institutions, the number increased to 21.3%. This greatly exceeds the separation rate of 10.7% for full-time nurses under normal circumstances.^[6]^ The responses indicated that in many cases, the reason for resigning was that “I cannot get support from my family.” Reportedly, 20.5% of nurses responded that they had experienced discrimination and prejudice. As context to this turnover, as might be expected, the intention to resign clearly exists,^[7]^ and it is believed that reducing the intention to resign leads to a reduction in turnover. This turnover activity negatively influences job performance and productivity,^[8,9]^ decreases the organizational commitment of nurses who continue working, increases burnout, and leads to a vicious cycle of more turnover.^[10]^ We consider searching for solutions to the problem of nurses leaving their jobs and preventing increases in the turnover rate extremely important during pandemics such as COVID-19. In general, turnover is associated with burnout, and the prevention of burnout is reportedly effective in reducing turnover among nurses.^[11,12]^ Outside of Japan, burnout has also been reported among healthcare workers involved with COVID-19 patients. For example, in Iran, the level of burnout syndromes among frontline nurses is higher than among other nurses, and the most important influencing factor is work stress.^[13]^ Surveys on burnout have also been conducted in Japan. In 1 study, more than 40% of nurses and 30% of radiology technicians and pharmacists reported symptoms that met the standard for burnout syndrome.^[14]^ However, we were unable to find literature on the association between intention to resign and psychiatric symptoms other than burnout.

Based on the above, the purpose of this study was to investigate the association between mental health (PTSD, depression, anxiety disorder, and burnout) and intention to resign, and influencing factors regarding nurses involved with COVID-19 patients in A Prefecture as subjects.

## 2 Research methods

### 2.1 Subjects

The subjects were nurses in A Prefecture working in a ward that cared for patients with a COVID-19 infection.

### 2.2 Study period

August 4–October 26, 2020.

### 2.3 Study design

A cross-sectional questionnaire-based study.

### 2.4 Study size

A letter explaining the study was mailed to the directors of nursing at 41 hospitals in A Prefecture, and the 5 hospitals from which consent was obtained were made the subjects of the research. In total, 120 surveys were mailed out, and responses were received from 56 people (7 males and 49 females) (response rate of 46.6%). The sample size was 56 people, the response rate was 46.6%, the confidence level was 90%, and the acceptable error was 10.9%. It is difficult to say that it was sufficient, but it was determined that there were no hindrances to analyzing the survey, as the numbers were tolerable.

### 2.5 Study description

To compare the findings of this study with those from China, the scale used by Jianbo Lai, Simeng Ma, Ying Wang, et al was used.^[4]^ Findings relating to burnout were added to the scale.

#### 2.5.1 Basic attributes

Gender, age, number of years of experience, place of work, marital status, whether or not they have children, etc.

As an influencing factor, perception of damage from harmful rumors was measured on a 5-point scale: “I do not feel it at all,” “I hardly feel it at all,” “I sometimes feel it,” and “I feel it a lot.” In addition, to study the effect of the number of COVID-19 patients, the survey was administered during the period in which the number of patients with COVID-19 peaked (August 5–September 5, 2020) and during the peak out period (September 6–October 26, 2020).

#### 2.5.2 Mental health factors

##### 2.5.2.1 Traumatic experience

The Japanese version of the internationally used Impact of Event Scale-Revised (IES-R) was used.^[15]^ Asukai, Kato, Kawamura, et al validated the reliability and suitability of the IES-R. The IES-R is an automatic recording questionnaire developed according to the US Diagnostic and Statistical Manual of Mental Disorders diagnostic standards, which measures posttraumatic stress symptoms. It is composed of 22 questions, 7 to Intrusion symptoms, 8 to avoidance symptoms, and 7 to hyper-arousal. Respondents evaluate the strength of the symptoms over the past week on a 5-point scale. IES-R scores are normal (0–8), mild (9–25), moderate (26–43), and severe (44–88). As a reference for screening the PTSD group, the cut-off point was 25 points or more for the PTSD high-risk group.

##### 2.5.2.2 Checking for Subthreshold depression

The Patient Health Questionnaire-9 (Japanese version of the PHQ-9) was developed by Kroenke, Spitzer, and Williams.^[16]^ This questionnaire is used to diagnose depression and evaluate the severity of symptoms. It was translated into Japanese,^[17]^ and its reliability and suitability have been validated. It comprises 9 questions, the responses to which concern respondents’ most recent 2 weeks, which are measured on a 3-point scale ranging from “0 = Never” to “3 = Almost every day.” The total score ranges from 0 to 27 points. Symptoms are evaluated as normal (0–4), mild (5–9), moderate (10–14), or severe (15–21). A score of 10 or more on the PHQ-9 meets the criteria for depression (major depression disorder); thus, this score was used in this study.

##### 2.5.2.3 Checking for anxiety

The Generalized Anxiety Disorder-7 (Japanese version of GAD-7) was developed by Spitzer, Kroenke, Williams, and Löwe.^[18]^ This questionnaire is used to evaluate generalized anxiety disorder. It was translated into Japanese,^[19]^ and its reliability and suitability have been validated. Responses to 7 questions about the evaluated symptoms are measured on a 3-point scale, where “0 = Never,” “1 = Several days,” “2 = More than half the time,” and “3 = Almost every day.” The total score ranges from 0 to 21 points. In the evaluation of symptoms, a score of 0 to 4 points indicates no anxiety disorder, 5 to 9 mild anxiety, 10 to 14 moderate anxiety, and 15 to 21 indicates severe anxiety disorder. A respondent who scores 10 points or more is considered a candidate for drug therapy.

##### 2.5.2.4 Checking for burnout

We used the Maslach Burnout Inventory-General Survey (MBI-GS).^[20,21]^ The MBI-GS was designed for use with occupational groups other than human services and education, including those working in jobs such as customer service, maintenance, manufacturing, management, and most other professions. The MBI-GS contains 3 scales: “exhaustion” (EX: 5 items), “cynicism” (CY: 5 items), and “professional efficacy” (PE: 6 items). EX measures feelings of being overextended and tired by one's work. CY measures indifference or a distant attitude toward one's work. PE measures satisfaction with past and present accomplishments; it explicitly assesses an individual's expectations of continued effectiveness at work. All items are scored on a 7-point Likert scale ranging from 0 (“never”) to 6 (“every day”). High EX and CY scores and low PE scores are indicative of burnout.

##### 2.5.2.5 Checking for intention to resign

According to the categories introduced by Tsuchie and Nakamura,^[22]^ presence or absence of Intention to leave the job including “wants to quit working as a nurse,” “wants to switch hospitals or departments,” and “wants to continue working as a nurse” are evaluated by 5 levels from “always present” to “absent.” The higher the scores, the stronger the participants’ will.

### 2.6 Procedures

- A questionnaire and QR code were created using the paid version of Google Forms.
- After the ethical review, the purpose of the research was explained to the directors of nursing of the hospitals, and consent was obtained.
- After obtaining approval from the directors of nursing, a questionnaire with a QR code and letter explaining the research were sent to each hospital, addressed to the nursing departments of each hospital.
- After the questionnaires arrived, the director of nursing explained to the nursing supervisors of each ward that a research request had been received.
- After the explanation from the director of nursing, the nursing supervisors of each ward explained the research request and asked for the survey request forms and survey forms to be distributed.
- Nurses who could participate in the survey received the abovementioned documents and responded to the survey using the QR code, which linked to the questionnaire.

### 2.7 Method of analysis

- The number and percentage of the base attributes of the subjects were calculated.
- Using scores from the PHQ-9, GAD-7, and ES-R scales, the subjects were classified as normal, mild, moderate, and severe.
- Using the MBI-GS classifications of Kitaoka, Masuda, Ogino, and Nakagawa^[23]^ as reference, the subjects were classified into 3 groups: “high,” “normal,” and “low.”
- To confirm the association between psychiatric symptoms (PTSD, depression, anxiety, burnout) and intention to resign, a multiple regression analysis was performed by the forced entry method.
- To determine the effect of the number of patients with COVID-19, the timing of the survey was investigated. Two time periods were employed: the period the number of COVID-19 patients peaked (August 5–September 5, 2020) and the peak out period (September 6–October 26, 2020).
- To identify the association between psychiatric symptoms and intention to resign during the peak and peak out periods, *t* tests were performed.
- Finally, to investigate the associations between perception of damage from harmful rumors, psychiatric symptoms, and intention to resign, a single-factor analysis of variance was performed using the following scale: “I do not feel it at all,” “I hardly feel it at all,” “I sometimes feel it,” and “I feel it a lot.” In addition, the Tukey method was employed for multiple comparisons.

### 2.8 Ethical considerations

This study was conducted after obtaining approval (No.: 20004) from the Hyogo University Ethical Review Board. Whenever research was conducted, a written research request was sent to the institution where the study was to take place. After obtaining approval, subjects received written explanations of the research. Only subjects from whom consent was obtained were included in the study. The written materials for the subjects explained that if they decided of their own free to not consent to participate in the research or to discontinue their participation, there would be no disadvantage to themselves and their data would not be used for any purpose other than for this study, that the data obtained would be processed statistically using code numbers, their privacy would be protected, and their personal information would not be disclosed.

## 3 Results

### 3.1 Basic attributes of the subjects

Regarding subjects’ basic attributes, 7 are males (12.5%) and 49 are females (87.5%) (Table 1). Most were aged 20 to 29 years (25 subjects, 44.6%) or 30 to 39 years (15 subjects, 26.8%). Most had 1 to 5 years of service (18 subjects, 32.1%) or 6 to 10 years (14 subjects, 25.0%). Regarding education, most had graduated from technical college (37 subjects, 66.1%), junior college (3 subjects, 5.4%), or university (12 subjects, 21.4%). The most frequent work formats were 3 shifts (25 subjects, 44.6%) or 2 shifts (20 subjects, 35.7%). Their rankings were staff nurses (48 subjects, 85.7%), supervisors (including assistant supervisors) (5 subjects, 8.9%), or nursing supervisors (including assistant nursing supervisors) (3 subjects, 5.4%). Furthermore, they were certified as nurses (56 subjects, 100%), public health nurses (6 subjects, 10.7%), or specialist nurse/certified nurses (3 subjects, 5.4%). They worked in the general wards (16 subjects, 28.6%), intensive care units (21 subjects, 37.5%), infectious disease wards (16 subjects, 28.6%), and pediatrics (3 subjects, 5.4%). For marital status, 20 subjects (35.7%) were married (including common-law marriage) and 36 subjects (64.3%) were unmarried.

**Table 1:** Basic attributes of the subjects.

		N	%			N	%
Gender	Males	7	12.5	Qualification	Nurses	56	100
	Females	49	87.5		Public health nurses	6	10.7
Age	20–29	25	44.6		specialist nurse/certified nurses	3	5.4
	30–39	15	26.8				
	40–49	9	16.1	Department	General wards	16	28.6
	50–59	7	12.5		Intensive care units	21	37.5
Years of service	1–5 yrs	18	32.1		Infectious disease wards	16	28.6
	6–10 yrs	14	25.0		Pediatrics	3	5.4
	11–15 yrs	6	10.7	Marital status	Married (including common-law marriage)	20	35.7
	16–20 yrs	5	8.9		Unmarried	36	64.3
	21–25 yrs	5	8.9	Work formats	Day shift	3	5.4
	26–30 yrs	4	7.1		Day shift and duty	5	8.9
	Over 30 yrs	4	7.1		3 shifts	25	44.6
Educational background	Technical college	37	66.1		2 shifts	20	35.7
	junior college	3	5.4		Night shift	3	5.4
	University	12	21.4	Their rankings	Staff nurses	48	85.7
	graduate School	2	3.6		Supervisors (including assistant supervisors)	5	8.9
	Advanced course	2	3.6		nursing supervisors (including assistant nursing supervisors)	3	5.4

### 3.2 Frequency of appearance of psychiatric symptoms (PTSD, depression, anxiety, burnout) and associations

Using the IES-R, PHQ-9, and GAD-7 scales, the subjects were classified in the normal, mild, moderate, and severe categories. In the resulting scores on the IES-R, 19 subjects (33.9%) were classified as normal, 23 (41.1%) as mild, 9 (16.1%) as moderate, and 5 (8.9%) as severe. Furthermore, 14 subjects (25.0%) in the group were identified as being at high risk for PTSD, who scored either moderate or severe. In the resulting scores on the PHQ-9, 30 subjects (53.6%) were classified as normal, 15 (26.8%) as mild, 7 (12.5%) as moderate, and 4 (7.2%) as severe. There were 11 subjects (19.7%) who scored in the moderate or severe categories, meeting the criteria for depression (major depression disorder). Based on the anxiety disorder scores, 30 subjects (53.6%) were classified as normal, 14 (25.0%) as mild, 8 (14.3%) as moderate, and 4 (7.1%) as severe. There were 12 subjects (21.4%) in the group who scored from moderate to severe, for which drug therapy is considered necessary (Table 2). In addition, using the MBI-GS classifications as a reference, subjects were classified into 3 groups: “high,” “normal,” and “low.” As a result, for symptoms of burnout, for “exhaustion,” 20 subjects (35.7%) scored low, 15 (26.8%) normal, and 21 (37.5%) high. For “cynicism,” 20 subjects (35.7%) scored low, 18 (32.1%) normal, and 18 (32.1%) high. Regarding “decrease in professional efficacy,” 33 subjects (58.9%) scored low, 14 (25.0%) normal, and 9 (16.1%) high (Table 3). About 30% or more of the nurses had high burnout.

**Table 2:** Frequency of appearance of psychiatric symptoms (PTSD, depression, anxiety) and associations.

	Severe		Moderate		Mild		Normal	
	N	%	N	%	N	%	N	%
PTSD	5	8.9	9	16.1	23	41.1	19	33.9
Depression	4	7.1	7	12.5	15	26.8	30	53.6
Anxiety	4	7.1	8	14.3	14	25.0	30	53.6

**Table 3:** Frequency of appearance of bournout and associations.

	High		Normal		Low	
	N	%	N	%	N	%
Burnout						
Exhaustion	21	37.5	15	26.8	20	35.7
Cynicism	18	32.1	18	32.1	20	35.7
Professional efficacy	9	16.1	14	25.0	33	58.9

### 3.3 Association between psychiatric symptoms (PTSD, depression, anxiety, burnout) and intention to resign

To confirm the relevance of psychiatric symptoms (PTSD, depression, anxiety, burnout), a multiple regression analysis was performed (Table 4). As a result, significant associations were confirmed between intention to resign (“I want to quit being a nurse”) with 2 symptoms of burnout, namely “cynicism” (β = 0.37, *P* < .01) and “decrease in professional efficacy” (β = −0.31, *P* < .01). Significant associations were also confirmed between “I want to change hospitals/wards” and the burnout symptom “cynicism” (β = 0.42, *P* < .01), and between “I want to continue working as a nurse” and subthreshold depression (β = −0.27, *P* < .05); anxiety disorder (β = −0.27, *P* < .05); and the burnout symptoms “exhaustion” (β = −0.41, *P* < .01), “cynicism” (β = −0.35, *P* < .01), and “decrease in professional efficacy” (β = 0.49, *P* < .01).

**Table 4:** Association between psychiatric symptoms (PTSD, depression, anxiety, burnout) and intention to resign.

	Intention to resign		
	I want to quit being a nurse	I want to change hospitals/wards	I want to continue working as a nurse
	β	β	β
PTSD			
PTSD	0.05	0.21	−0.11
Intrusion symptoms	0.09	0.26	−0.04
Avoidance symptoms	0.06	0.15	−0.15
hyper-arousal	0.03	0.17	−0.12
Depression			
Depression	0.01	0.16	−0.27^∗^
Anxiety			
Anxiety	0.01	0.27	−0.27^∗^
Burnout			
Exhaustion	0.22	0.08	−0.41^†^
Cynicism	0.37^†^	0.42^†^	−0.35^†^
Professional efficacy	−0.31^†^	0.01	0.49^†^
R^2^	0.34	0.27	0.53

### 3.4 Comparison of psychiatric symptoms and intention to resign in the period the number of COVID-19 patients peaked (August 5–September 5, 2020) and peak out period (September 6–October 26, 2020)

To study the association between psychiatric symptoms and intention to resign during the peak of the second wave of COVID-19 and peak out period in Japan, *t* tests were performed (Table 5). The results indicated that anxiety disorder was significantly higher during the peak period (*P* < .05). Furthermore, for intention to resign, significant differences were confirmed between “I want to quit being a nurse” (*P* < .01), “I want to change hospitals/wards” (*P* < .05), and “I want to continue working as a nurse” (*P* < .05). In other words, the results indicated that when the number of COVID-19 patients increased, both nurses’ anxiety and their intention to resign increased as well.

**Table 5:** Comparison of psychiatric symptoms and intention to resign in the period the number of COVID-19 patients peaked (August 5–September 5, 2020) and peak out period (September 6–October 26, 2020).

	Peak period (N = 44)		Peak out Period (N = 12)			
	M	SD	M	SD	t	*P*
PTSD						
PTSD	19.09	17.16	14.27	12.82	1.03	n.s.
Intrusion symptoms	7.68	6.72	5.58	4.27	1.02	n.s.
Avoidance symptoms	6.11	6.23	4.08	5.20	1.03	n.s.
hyper-arousal	5.35	4.82	4.09	4.25	0.85	n.s.
Depression						
Depression	6.43	5.37	4.08	4.29	1.56	n.s.
Anxiety						
Anxiety	5.93	5.13	3.18	4.29	1.82	^∗^
Burnout						
Exhaustion	3.32	1.69	2.55	1.79	1.38	n.s.
Cynicism	2.05	1.35	1.30	1.56	1.63	n.s.
Professional efficacy	1.76	1.27	1.92	1.48	−0.37	n.s.
Intention to Resign						
I want to quit being a nurse	3.20	1.05	2.08	1.24	3.16	^†^
I want to change hospitals/wards	3.16	1.41	2.33	1.37	1.81	^∗^
I want to continue working as a nurse	3.09	1.16	3.83	1.11	−1.98	^∗^

### 3.5 Comparison of presence of perception of damage from harmful rumors and psychiatric symptoms and intention to resign

To study the associations between perception of damage from harmful rumors, psychiatric symptoms, and intention to resign, a single factor analysis of variance was performed (Table 6). Furthermore, the Tukey method was used for multiple comparisons. To the question about whether they felt damage from harmful rumors, the responses were “I do not feel it at all” (N = 7), “I hardly feel it at all” (N = 25), “I sometimes feel it” (N = 16), and “I feel it a lot” (N = 6), indicating that damage was felt. Significant differences were confirmed between “PTSD” (*P* < .05) and the PTSD symptoms “intrusive symptoms” (*P* < .05), “avoidance symptoms” (*P* < .05), “hyper-arousal” (*P* < .05), “subthreshold depression” (*P* < .05), “anxiety disorder” (*P* < .05), and “cynicism” on the burnout scale (*P* < .05). The results of the multiple comparisons indicated that for damage from harmful rumors, “I feel it a lot” was more significant than “I do not feel it at all.”

**Table 6:** Comparison of presence of perception of damage from harmful rumors and psychiatric symptoms and intention to resign.

	I do not feel it at all (N = 7)		I hardly feel it at all (N = 25)		I sometimes feel it (N = 16)		I feel it a lot (N = 6)			
	M	SD	M	SD	M	SD	M	SD	F	*P*
PTSD										
PTSD	5.29	5.25	17.44	15.02	19.31	13.34	32.67	26.49	3.48	^∗^
Intrusion symptoms	2.57	2.15	6.92	5.86	7.59	5.43	13.00	9.70	3.38	^∗^
Avoidance symptoms	1.43	2.94	5.96	5.45	5.18	5.09	10.83	10.05	2.96	^∗^
hyper-arousal	1.29	1.38	4.68	4.16	6.00	4.35	8.83	7.19	3.49	^†^
Depression										
Depression	1.86	1.95	5.44	5.59	8.53	4.87	5.80	3.56	3.10	^∗^
Anxiety										
Anxiety	0.57	0.79	4.79	4.32	8.06	5.62	5.67	4.97	4.59	^†^
Burnout										
Exhaustion	9.86	7.90	16.19	7.75	16.59	9.43	18.50	9.89	1.40	n.s.
Cynicism	3.86	3.76	9.12	6.99	10.19	7.82	15.17	3.92	3.15	^∗^
Professional efficacy	11.71	12.91	10.28	7.59	9.31	6.57	12.80	3.96	0.32	n.s.
Intention to Resign										
I want to quit being a nurse	2.86	1.21	2.65	1.23	3.18	0.95	3.83	1.17	2.01	n.s.
I want to change hospitals/wards	3.29	1.38	2.88	1.45	2.71	1.45	3.83	1.33	1.06	n.s.
I want to continue working as a nurse	3.86	1.07	3.42	1.17	2.82	1.13	3.00	1.26	1.70	n.s.

## 4 Discussion

### 4.1 Frequency of appearance of psychiatric symptoms (PTSD, depression, anxiety, burnout)

In the group, 14 subjects (25.0%) were identified as being at high risk for PTSD, scoring either moderate or severe. Furthermore, 11 subjects (19.7%) scored moderate or severe, meeting the criteria for depression (major depression disorder). In addition, 12 subjects (21.4%) scored from moderate to severe for anxiety disorder, for which drug therapy is considered necessary, and about 30% or more of the nurses had high burnout. Regarding PTSD, of the respondents in a study by Jianbo Lai, Simeng Ma, Ying Wang, et al,^[4]^ 22.5% were classified as normal, 38.2% as mild, 24.6% as moderate, and 11.6% as severe. Compared to China, this survey had more subjects who scored in the normal and mild categories. For subthreshold depression, of the respondents in Jianbo Lai et al,^[4]^ 46.5% were classified as normal, 38.1% as mild, 8.4% as moderate, and 7.1% as severe. Compared to China, our study had more subjects classified as moderate and about the same number categorized as severe. Regarding anxiety disorder, of the respondents in Lai et al,^[4]^ 52.9% were classified as normal, 34.4% as mild, 7.1% as moderate, and 5.6% as severe. Compared to China, more subjects in our study were classified in the severe and moderate categories. In other words, in Japan, working as a nurse caring for patients with COVID-19 increased PTSD, subthreshold depression, and anxiety disorders. These results support the findings of Sampaio, Sequeira, and Teixeira.^[5]^ This suggests the importance of the mental health of nurses during the COVID-19 pandemic in Japan as well. As evidence of the worldwide effect of the COVID-19 pandemic on mental health, sleep disorders, anxiety, and depression have been reported among healthcare workers and other vulnerable groups.^[24]^ The same is true for nurses in Japan. For nurses, the fear of being infected, the critical lack of resources, social isolation, large-scale financial losses, and the huge amount of uncertainty affect a wide range of psychological distresses, and are believed to amplify the risk of psychiatric illness and behavior disorders as a result of COVID-19.^[25]^

Next, the results indicate that at least 30% of nurses are experiencing high levels of burnout. Consistent with Matsuo et al,^[14]^ this study also found that around 40% of nurses are burned out. These results further support those of Sarboozi et al.^[13]^ Nurses take care of COVID-19 patients at the frontlines, and the prospects for the future are unclear. We believe these circumstances increase the risk of burnout.

### 4.2 Association between psychiatric symptoms (PTSD, depression, anxiety, burnout) and intention to resign

The correlation between “I want to continue working as a nurse” under the category of resignation of nurses and psychiatric disorders was confirmed. An association was confirmed between “I want to quit being a nurse” with “cynicism” and “decrease in professional efficacy” under burnout. “I want to change hospitals/wards” was only associated with “cynicism” under burnout. Furthermore, a significant association was confirmed between “I want to continue working as a nurse” and “subthreshold depression,” “anxiety disorder,” “exhaustion,” “cynicism,” and “decrease in professional efficacy” under burnout. According to the results of Ohue et al,^[11]^ a strong association exists between each of the burnout symptoms and intention to resign. They found strong associations between “I want to quit being a nurse” and “exhaustion,” “cynicism,” and “professional efficacy.” However, our survey only found associations with “cynicism” and “professional efficacy.” A strong association with “I want to continue working as a nurse” was also confirmed. We believe these are special characteristics of nurses involved with COVID-19 patients. In other words, for “exhaustion,” looking after patients infected with COVID-19 is a mission for nurses, and they do not think they “want to quit being a nurse.” Rather, when the “exhaustion” continues and progresses to a decrease in “cynicism” and “professional efficacy,” “I want to quit being a nurse” arises. Furthermore, it is thought that mental health distress manifests in a decrease in “I want to continue working as a nurse” rather than “I want to quit being a nurse.” Nurses with a strong sense of mission continue to be involved with patients with COVID-19. Those engaged in caring for these patients are in a state of mental distress. The fact that the intention to resign is increasing is a sign that intervention for nurses is essential. Ohue et al^[12]^ reported on the efficacy of cognitive behavioral therapy as a way to prevent burnout among nurses. Possibly, using a psychological support method such as cognitive behavioral therapy might maintain nurses’ mental health and be able to prevent turnover during a pandemic.

### 4.3 Association of psychiatric symptoms and intention to resign in the period numbers of COVID-19 patients peaked (August 5–September 5, 2020) and the peak out period (September 6–October 26, 2020)

When the association between psychiatric symptoms and intention to resign during the peak of the second wave of COVID-19 and peak out period in Japan was studied, a significant association was confirmed between anxiety disorder and intention to resign: “I want to quit being a nurse,” “I want to change hospitals/wards,” and “I want to continue working as a nurse” during the peak. In other words, the results indicated that when the number of COVID-19 patients increased, both nurses’ anxiety and intention to resign increased. In addition, although no significant difference was confirmed, the average values tended to be higher at the peak. The peak period between August 5 and September 5, 2020 corresponds to what is called the second wave in Japan. It came shortly after the lifting of the Emergency Declaration in the first wave. In addition, during the time the infection spread, the workload and working hours of healthcare workers tasked with preventing infection in conjunction with an increase in infected patients became harder and longer. Furthermore, the fear of becoming infected became a large source of stress for them. Thus, we believe that as the number of patients increased, so too did the amount of anxiety and intention to resign. Therefore, we believe that reducing the patient load is critical for improving the mental health of nurses.

### 4.4 Associations between the presence of perception of damage from harmful rumors and psychiatric symptoms and intention to resign

The associations between perception of damage from harmful rumors and psychiatric symptoms, and intention to resign were studied. To the question about whether they felt damage from harmful rumors, the responses were: “I do not feel it at all” (N = 7), “I hardly feel it at all” (N = 2), “I sometimes feel it” (N = 16), and “I feel it a lot” (N = 6), indicating that damage was felt. The results indicate that nurses who felt damage from harmful rumors experienced high amounts of the PTSD symptoms “intrusive symptoms,” “avoidance symptoms,” “hyper-arousal,” “subthreshold depression,” and “anxiety disorder,” as well as the burnout symptom “cynicism.” The Japan Medical Association compiled a report on damage from harmful rumors to healthcare workers involved with patients infected with COVID-19.^[6]^ It reported that healthcare workers other than physicians, particularly nurses, were harmed. Furthermore, the Japanese Nursing Association reported that the burden on healthcare workers at the frontlines of medicine is extremely high, and that the addition of discrimination and prejudice worsens the physical and emotional exhaustion. The report also stated that this would lead to the turnover of healthcare workers and increase the risk of the healthcare system breaking down. This research underscores this view.^[6]^

Prejudice and damaging rumors against healthcare staff involved with COVID-19 patients in Japan are acknowledged as a problem. Originally, damage from harmful rumors was defined as “the economic damage caused by consumers seeing foodstuffs, products, land, or people ordinarily considered safe as dangerous, and stopping consumption or travel, due to large-scale media coverage of certain incidents, accidents, environmental pollution, or disasters.”^[26]^ According to Sekiya,^[26]^ as the term suggests, damage from harmful rumors is not caused by “rumors” or “baseless rumors,” but based on a fact that acts as a trigger from which the rumors grow. It is believed that the cause of the emergence of harmful rumors is that COVID-19 is intangible and generates anxiety. Matsui compiled overall views from the aid activities and research of psychologists in the aftermath of the Great East Japan Earthquake Disaster.^[27]^ It is thought that the damage caused by the harmful rumors in conjunction with the Great East Japan Earthquake increased because of anxiety about the effects of radiation and living a certain distance from the devastated area. However, this was controlled by providing accurate and correct knowledge and attitudes regarding making rational judgments. Shigemura, Takahashi, Oe, and Kurosawa stated that understanding the psychosocial reaction to COVID-19 as “reacting to a potential disaster that is not visible to the eye,” rather than as “reacting with suspicion” contributes to reducing anxiety.^[28]^ In other words, since COVID-19 is invisible, it brings a variety of anxieties to the forefront, but damage from harmful rumors must be controlled by recognizing that they are a “reaction to a potential disaster that is not visible to the eye.” Furthermore, providing the correct information is necessary. We believe this can contribute to improving the mental health of nurses.

### 4.5 Limitations of this study and future challenges

The main limitation of this study was the small size of the sample. We attribute the small sample size to the difficulty in obtaining the cooperation for research data collection of hospitals that accepted patients with COVID-19. Possibly, perhaps the lack of cooperation was to protect the hospitals. Because of this, we conducted our study using limited statistical methods. In the future, it will be necessary to find a way to increase the number of samples. In addition, the research was limited to A Prefecture because of the regional disparity in COVID-19 cases. In the future, different regions should be surveyed. Finally, based on the data in this study, future research should aim to build a mental health support system for nurses in times of a pandemic and create a community to prevent damage from harmful rumors. To reduce nurse turnover in a pandemic, it is necessary to construct mental health support systems and a community support system for preventing the spread of false rumors in ordinary times. These actions are critical for preventing the collapse of the healthcare system during a pandemic.

## 5 Conclusion

The purpose of this study was to investigate the association between the mental health (PTSD, depression, anxiety disorder, and burnout) of nurses involved with patients with COVID-19 and job turnover. The subjects of the study were nurses working in wards charged with caring for patients with COVID-19 in A Prefecture. An additional goal was to investigate the influencing factors. Based on our results, we draw the following conclusions.

1. Between 20% and 30% of nurses involved with patients with COVID-19 are in a state of high mental distress.
2. Regarding the associations between psychiatric symptoms and intention to resign, “I want to quit being a nurse” was affected by “cynicism” and “professional efficacy”; “I want to change hospitals/wards” was affected by “cynicism”; and “subthreshold depression,” “anxiety disorder,” and “burnout” affected “I want to continue working as a nurse.”
3. The increase in the number of patients with COVID-19 was a factor affecting mental health and intention to resign. When the number of patients increased, anxiety disorders and intention to resign also increased. Damage from harmful rumors increased the severity of every psychiatric symptom.
4. To prepare for a pandemic such as COVID-19, it is necessary in normal times to construct psychological support systems and community systems to prevent damage from harmful rumors.

## Acknowledgments

The authors express their gratitude to all the nursing directors and nurses who cooperated with them despite the burdens of the coronavirus crisis.

## Author contributions

Contributors Takashi Ohue was responsible for the organisation and coordination of the trial. Takashi Ohue was the chief investigator and also responsible for the data analysis. Takashi Ohue, Eiichi Tougo, Yuka Ohue, Kazuko Mitoku contributed to the writing of the final manuscript. All members of the Study Team contributed to the management or administration of the study.

**Conceptualization:** Takashi Ohue.

**Data curation:** Takashi Ohue, Eiichi Tougo, Yuka Ohue, Kazuko Mitoku.

**Formal analysis:** Takashi Ohue.

**Funding acquisition:** Takashi Ohue.

**Investigation:** Takashi Ohue.

**Methodology:** Takashi Ohue.

**Project administration:** Takashi Ohue.

**Resources:** Takashi Ohue.

**Software:** Takashi Ohue.

**Supervision:** Takashi Ohue.

**Validation:** Takashi Ohue.

**Visualization:** Takashi Ohue.

**Writing – original draft:** Takashi Ohue.

**Writing – review & editing:** Takashi Ohue.
